# Ventilators, Settings, Autotitration Algorithms

**DOI:** 10.3390/jcm12082942

**Published:** 2023-04-18

**Authors:** Manel Luján, Cristina Lalmolda

**Affiliations:** 1 Servei de Pneumologia, Hospital Universitari Parc Taulí, 08208 Sabadell, Spain; 2 Centro de Investigacion Biomédica en Red (CIBERES), 28029 Madrid, Spain

**Keywords:** trigger, pressurization, ventilatory modes, hybrid modes, upper airway obstruction, expiratory flow limitation

## Abstract

The choice of a ventilator model for a single patient is usually based on parameters such as size (portability), presence or absence of battery and ventilatory modes. However, there are many details within each ventilator model about triggering, pressurisation or autotitration algorithms that may go unnoticed, but may be important or may justify some drawbacks that may occur during their use in individual patients. This review is intended to emphasize these differences. Guidance is also provided on the operation of autotitration algorithms, in which the ventilator is able to take decisions based on a measured or estimated parameter. It is important to know how they work and their potential sources of error. Current evidence on their use is also provided.

## 1. Introduction

The precursors of home NIV date back to the polio epidemics in the 1950s, which gave rise to the first devices, mainly ventilators, using negative extrathoracic pressure as a method of rib cage expansion (iron lung) [1]. However, these early models presented some important problems, such as the difficulty of transport and the possibility of side effects during their use, such as upper airway obstructive events. Later, in 1984, intermittent positive pressure was introduced for patients with muscular dystrophies and, since then, it has become the treatment of choice for both acute and chronic respiratory failure secondary to various diseases [2,3].

Over the last two decades, the performance and improvements of NIV devices have progressively evolved in available ventilatory modes, range of set parameters and monitoring capabilities. Classically, older devices were able to work in a single ventilation mode (volumetric or barometric) and were not equipped with monitoring capabilities beyond a rudimentary counter to control compliance. All positive pressure devices can be classified according to different criteria, as each country has its own legislation, which classifies ventilators according to the situation in which they are indicated for NIV (life support, need for a battery, etc.) [4]. From a more generic perspective, the most agreed classification classifies them as bi-level, intermediate respiratory care ventilators and ICU ventilators. Bi-level ventilators operate with a turbine system, single limb circuit and intentional leakage. They are usually transportable, have a good number of alarms and some have an internal battery. They are mainly used in chronic ventilation. ICU ventilators were primarily intended for invasive ventilation, although more modern ventilators have added “NIV modes”, which include a leak compensation system. They are usually operated with dual-limb systems and the operating mechanism can be either piston or compressor driven. They are equipped with a sophisticated alarm system and their use is restricted to critical care units. Finally, intermediate-care ventilators fall between the two categories mentioned above. They are usually equipped with an internal battery and are multifunctional, being used from the patient’s home to medical transport or emergency units [5,6].

However, the use of home ventilators to treat acute situations is not uncommon, although explicitly discouraged in a recent consensus document [7].

One of the first decisions about NIV is the choice of ventilator. The clinician usually takes his or her choice based on characteristics such as the transportability of the device, the existence or not of a long-lasting internal battery, the variety of ventilatory modes available (including simple modes and autotitration algorithms) or the possibility of additional monitoring. Concerning this latter point, nearly all home NIV ventilators are currently fitted with the so-called “built in software” technology that provides data on unintentional leaks, respiratory rate, tidal volume, compliance or upper airway events, even displaying cycle-by-cycle flow and pressure waveforms with reasonable reliability and detail [8]. These ventilators are also equipped with electronic alarm systems that can alert about potential problems during ventilator use, improving the safety of the devices. Most of these alarm systems are adjustable, but in clinical practice, their adjustment needs to be carefully balanced to maintain minimum safety while at the same time not causing unnecessary alarms that affect the quality of sleep of the ventilated patients.

## 2. Overview of Ventilatory Modes and Settings

In each mode, there is always a control or independent variable, which is programmed into the ventilator and remains constant throughout the inspiratory cycle, regardless of the variability in the patient’s ventilatory pattern. The control variables are usually pressure and volume. However, there are special cases, such as hybrid modes (average volume-assured pressure support -AVAPS- or intelligent volume-assured pressure support—iVAPS-) in which the control variable (pressure) is modified in a predetermined pressure range depending on the estimation of a parameter (tidal volume), see Section 3 [9]. Thus, ventilatory modes can be categorized into volume-targeted ventilation and pressure-targeted ventilation [10]. The pressure-targeted mode will be discussed in more detail as it is the currently preferred mode [11].

### 2.1. Volume-Limited or Volumetric Mode

The programmed volume remains constant as the independent variable and the pressure (dependent variable) changes depending on lung compliance, set volume, airway resistance and the patient’s inspiratory effort [12]. The main advantage of this ventilatory mode is that the volume is always delivered, regardless of airway resistance and lung compliance. The main drawbacks would be the efficiency loss in presence of leaks, as it does not compensate for them and the poor response in front of an increased patient’s effort (the delivered volume will not increase) [13]. Classically, it was the most widely used mode at the beginning of the home NIV era but has now been overshadowed by the pressure modes. The main parameters within this ventilatory mode are:

- The tidal volume (VT) or volume delivered in each ventilatory cycle. In NIV, this is usually set at around 8–10 mL/kg of ideal patient weight to overcome the effect of potential leaks.
- The basal respiratory rate (RR), is usually programmed 2–4 cycles below the patient’s spontaneous rate.
- Inspiratory time (Ti). It should be noted that cycling (transition from inspiration to expiration) is always based on a time criterion in volumetric modes. A shorter Ti is usually used in patients with obstructive lung mechanics and a longer Ti in restrictive ones.
- The shape of the flow waveform, which can be constant (flow is the same throughout the inspiratory cycle) or decelerating, more physiological, or with a higher flow at the beginning of inspiration.
- The level of positive end-expiratory pressure (PEEP).
- The trigger sensitivity.

### 2.2. Pressure-Limited or Barometric Mode

This is currently the most widely used ventilatory mode, due to its ability to compensate for leaks and its physiological mechanism, which allows the patient to maintain some control over tidal volume and inspiratory time (the latter in pressure support mode only) [13]. There are two main variants, pressure control (PC) and pressure support (PS). Both are based on a pre-set constant positive pressure at two different levels, inspiratory and expiratory, named Inspiratory positive airway pressure (IPAP) and Expiratory positive airway pressure (EPAP). The difference or gradient of pressures between the two is called “pressure support” (PS). The volume delivered in this ventilatory mode will depend, in addition to the pressure support gradient, on the patient’s lung mechanics and effort, which makes this mode more physiological [13]. These devices work by means of a turbine that will provide the necessary flow to reach the pre-set inspiratory and expiratory pressure values.

Moreover, some additional modes based on the possibility of a set backup respiratory rate can be found: assisted (or spontaneous “S”) where there is no safety backup respiratory rate; assisted with a backup respiratory rate (Spontaneous/timed “S/T”) or assisted-controlled (A/C) where there is a programmed safety frequency and in the event that the patient’s spontaneous frequency is lower than this, the ventilator starts to provide the cycles that will cycle by time in A/C and by flow in S/T and controlled (C).

Contrarily to volume-limited modes, an important feature of pressure modes that makes them particularly suitable for delivery as home NIV is their ability to compensate for moderate leaks. Additionally, patient-ventilator synchrony is usually much better in pressure-limited modes. The main drawback of pressure-limited modes is that they do not ensure a specific tidal volume (except for hybrid modes). Thus, the tidal volume will depend on the programmed pressure support (higher PS, higher VT), the impedance of the patient’s respiratory system (resistance and compliance) and the magnitude of the patient’s inspiratory effort. The main settings that can be modified in a barometric mode in a standard ventilator would be the following:

#### 2.2.1. IPAP and EPAP Levels

The absolute values of both pressures will depend on several conditions: for example, EPAP level is usually set at a minimum pressure of 4 cm H_2_O, to avoid rebreathing if a single limb with intentional leakage is used. If a double limb or a single limb with an active valve is used, the addition of EPAP is not strictly necessary. In addition, EPAP can be increased in certain conditions, such as upper airway obstructions in patients with obstructive apnea syndrome or expiratory flow limitation (EFL) in patients with COPD or obesity.

The IPAP level is usually set based on tidal volume monitoring. A value of around 7–8 mL/kg of ideal body weight is usually taken as a reference.

#### 2.2.2. Backup Respiratory Rate (BURR)

BURR is defined as the number of controlled breaths delivered by the ventilator in one minute to cope with an eventual drop in the patient’s RR in the absence of patient effort. It is usually set 2–4 cycles/min below the patient’s spontaneous RF, as in volumetric modes. The lack of BURR was associated with an increased number of upper airway events, mixed and central, in patients with obesity-hypoventilation syndrome [14]. There are differences between manufacturers regarding the cycling criteria in controlled cycles when pressure support is used. In most models, the transition criterion from inspiration to expiration is time, although some manufacturers maintain the flow criterion for both assisted and controlled breaths. Finally, some hybrid modes, such iVAPS and AVAPS have auto backup rates with iVAPS targeting 2/3 of the set rate and increasing during central apnea and AVAPS based on recent breathing patterns (only in automatic mode) [9].

#### 2.2.3. Trigger Sensitivity

It corresponds to the effort level at which a cycle is delivered in response to the patient’s demand, and controlling the transition from EPAP to IPAP. It is usually indicated as a numerical value in L/min or in an ordinal scale, corresponding the lower values to the most sensitive levels. It is a crucial parameter to ensure patient-ventilator synchronization. Since the introduction of trigger mechanisms 30 years ago, ventilator technology has improved, so the effort required by the patient to obtain an assisted cycle is considerably less in modern ventilators compared to those of the last decade of the last century. This technological progression has been accompanied by a redesign of the trigger variable (from older pressure trigger designs to newer sophisticated electronic trigger systems). As reflected in the review by Sinderby [15], the parameters that are usually taken as a reference for trigger designs are not measured directly but are the indirect consequences of the patient’s ventilatory drive in the circuit. Thus, pressure triggers react to a depressurization in the circuit because of patient effort and flow triggers react to increases in this parameter measured inside the ventilator. Only the NAVA (Neural Adjusted ventilatory assist) system uses a parameter directly measured in the patient, such as diaphragmatic electromyography, but its invasiveness (it requires placement of a nasogastric tube equipped with electromyographic sensors) and its high cost make it impractical to implement as a mode of home NIV [16].

Early ventilator models used the pressure trigger, in which the patient’s effort in front of a closed valve decreases the pressure in the circuit below a pre-set threshold (sensitivity) to receive the ventilator-assisted cycle. Apart from the decreased sensitivity compared with newer designs, the main drawback of the pressure trigger was the presence of nonintentional leakage, since the patient’s effort needs to be higher to compensate for these leaks and at the same time enough to decrease the pressure inside the limb [17].

**Figure 1:**
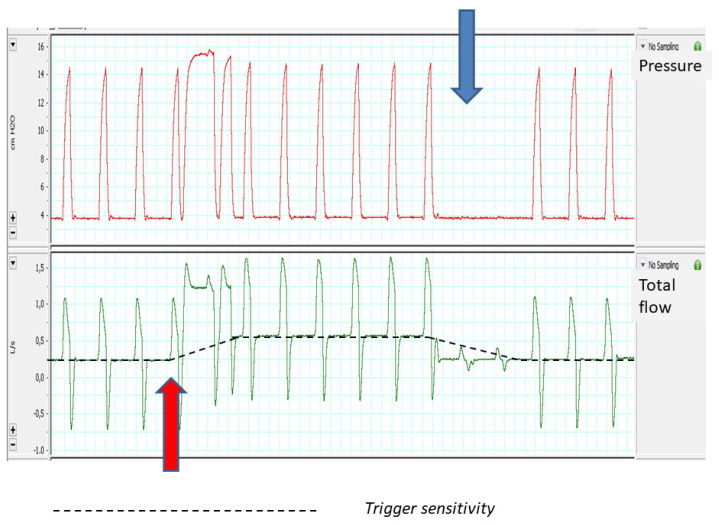
Effects of leakage on flow triggering function (see the text for more details).

The most common trigger system in modern ventilators is the flow trigger, in which the ventilator recognizes changes in the basal flow provided by the ventilator, with these changes interpreted as a patient’s ventilatory demand. These systems have shown higher sensitivity than older pressure trigger models [18]. However, the use of fixed-value flow thresholds to trigger the ventilator may be a problem in the presence of leaks: in the case that the leak compensation flow provided by the ventilator to keep the set pressure constant reaches the trigger threshold, it may be misinterpreted as a patient effort, delivering non-demanded assisted cycles (auto-triggering). For this reason, flow trigger systems should be accompanied by an algorithm to automatically adjust their sensitivity based on estimated baseline leakage (increasing threshold -and thus decreasing sensitivity-) [19]. These algorithms work independently of the preset sensitivity level on the ventilator and may lead to certain paradoxical effects: for example, if the sensitivity level is automatically decreased in presence of leaks, the patient’s effort needs to be higher to trigger the ventilator, leading eventually to ineffective efforts. Figure 1 shows an example in a bench experiment of automatic adjustments of the flow trigger during a short period of leakage introduction. As can be seen in this figure, the introduction of leakage (red arrow) induces immediate autotriggering, which corrects spontaneously after a few cycles, when the ventilator has set the new trigger sensitivity. Inversely, when the leakage was withdrawn (blue arrow), again the ventilator takes some breaths to re-adjust trigger sensitivity, favoring ineffective efforts during this brief time lapse (post leakage ineffective efforts). It seems clear that, in ventilators dedicated to NIV, the trigger should be considered a dynamic parameter, with fluctuations of the sensitivity level depending on the events that may occur during ventilator use, mainly leaks. Thus, there are differences in ventilator response to the introduction of external leaks in bench studies that can only be justified from the point of view of different trigger sensitivity algorithms [20].

Carteaux et al. [21] studied the performance of 19 ventilators under three conditions: no leak, continuous leak and inspiratory leak (using an underwater column). They found significant differences in the presence of trigger delay and autotriggering if the ventilators studied incorporated a specific non-invasive ventilation algorithm or not. Autotriggering was not observed in any of the NIV-specific ventilators. In contrast, Ferreira et al. [20], also on a bench test, found that most of the ventilators studied (specific to critical and acute non-invasive ventilation) required additional manual adjustments to avoid the presence of asynchronies in presence of leakage. A good surrogate for anticipating asynchronies is the increased work required to activate the mechanism in the presence of leaks (pressure-time product for the trigger -PTPtrig-), which would be in line with the effects of auto-adjusting algorithms, which decrease the sensitivity of the trigger in the presence of leaks [22].

**Figure 2:**
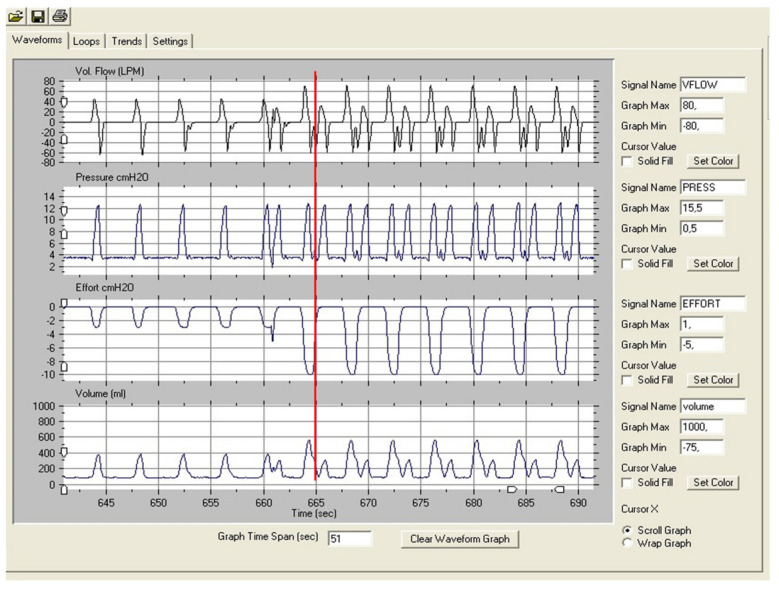
Autotriggering induced by noise in the expiratory phase of the cycle in a ventilator with electronic trigger (shape method). Observe that it cannot be considered as a double triggering because the effort (red vertical line) clearly ended before the start of the second cycle.

Finally, trying to compensate for these drawbacks in the flow-based trigger designs, alternative but more complex systems have appeared, such as the Auto-trak^®^ system (Philips Respironics^®^) based on the crossover points generated by a delayed virtual waveform superimposed (flow waveform method) on the native one [23]. In a clinical study of trigger sensitivity comparing flow-based and shape methods, it was demonstrated that despite the shape method being more sensitive (less ineffective efforts) it was at the same time less specific (more auto-triggering). A good example of the inappropriate reaction of this trigger system in front of any kind of noise is provided in Figure 2. In this bench test, the use of high efforts caused a “noise” during expiratory effort return to the baseline, causing autotriggering in the electronic trigger [24]. However, in contrast, in a bench to bedside study, Lalmolda et al. demonstrated that in front of the external gas introduction in the limb, the Auto-trak system was reasonably stable [25].

Another complex trigger system is the so-called “Energy trigger” (Breas), based on the calculation of the first flow derivative [26]. In a bench study combining leakage and simulated obstruction, Zhu et al. [27] already demonstrated that in a ventilator with the same Energy trigger model (Vivo 60^®^) the critical leakage level for triggering asynchronies was lower than in other ventilators.

In conclusion, the set of design differences, together with the manufacturer-specific trigger sensitivity levels, may account for the individual behaviors in the ventilators. It appears that flow triggers are less sensitive, mainly in the presence of leakage and probably because of the auto-adjusting sensitivity algorithm, while complex trigger systems tend to be more sensitive but may also be less specific.

#### 2.2.4. Pressurization Ramp (“Rise Time”)

From a conceptual point of view, the ramp is the parameter that controls the time between the start of the inspiratory cycle and the point at which the prescribed IPAP is reached. The ramp or rise time can be set based on a time scale (usually ms) or in a numerical analogic scale, corresponding usually to the lower numbers to the fastest ramp and shortest time values. At the same time, these shortest time levels will also correspond to the highest flow values, so the ramp will be closely linked to the inspiratory muscle unloading. In the acute patient mainly shorter values should be set, whereas, in chronic home NIV, shorter values should be mainly reserved for the obstructive patient and longer values for the restrictive one. Typical values are between 50 and 500 ms. It should be noted that the rise time will influence the cycling sensitivity depending on whether the peak inspiratory flow is reached earlier or not.

However, the concept of pressurization ramp should be analyzed in-depth, since it was classically considered as a “time-set”. In other words, the time to reach IPAP was constant for each ramp level, irrespective of changes in pulmonary or rib cage mechanics or increases in the patient’s effort. Battisti et al. [28] studied 10 home ventilators in a bench model, setting two different pressure levels on the ventilators and four active effort levels on an active simulator. In this study, significant differences in ventilator response to increasing effort and leakage were also found in the pressure-time product (PTP) at 300 ms, Similar results were found in a group of intermediate respiratory care ventilators [29]. These differences are hard to explain if the ramp was time-limited.

Lalmolda et al. conducted a bench to-bedside study for evaluating the pressurization capabilities of nine different ventilators, two for the acute care setting and seven for home ventilation. They found important differences among studied ventilators in PTP300. In addition, the bedside study focused on COPD patients and used parasternal EMG as a surrogate of inspiratory muscle unloading, showed that the ventilators with worse performance in bench tests showed less muscle unloading at the same pressure support level. Finally, these authors concluded that the parameter controlling the ramp seems not to be the time, but the flow changes (mathematically, the first derivative of the flow) [30].

#### 2.2.5. Cycling to Expiration

Cycling to expiration or expiratory trigger is related to the criterion used by the ventilators to control the transition from the inspiratory to expiratory phase. These criteria are a percentage of peak (maximum) flow in pressure support mode and a fixed inspiratory time in pressure control mode. In PS mode, an expiratory trigger can be set directly as a percentage of peak inspiratory flow or in a numerical (1–9) or nominal scale (sensitive, medium, low sensitive) depending on the manufacturer. In the first case (direct setting of the percentage of peak flow) it is usually set high if a short inspiratory time is desired (e.g., in COPD patients, where a short inspiratory time/total time ratio is desirable) and lower if a longer inspiratory time is desired (e.g., in restrictive patients). If a numerical scale is used, the lowest values (1 to 3) usually correspond to the highest percentages with respect to peak flow. Finally, on the nominal scale, the term “sensitive” is also related to the highest percentages relative to peak flow, and therefore, to the shortest inspiratory time values.

Some devices are equipped with more sophisticated systems, such as the “Auto-trak™ Respironics” which detects the patient’s breathing pattern based on an imaginary waveform and automatically adjusts the trigger sensitivity and cycling thresholds.

#### 2.2.6. Maximum and Minimum Inspiratory Time

**Figure 3:**
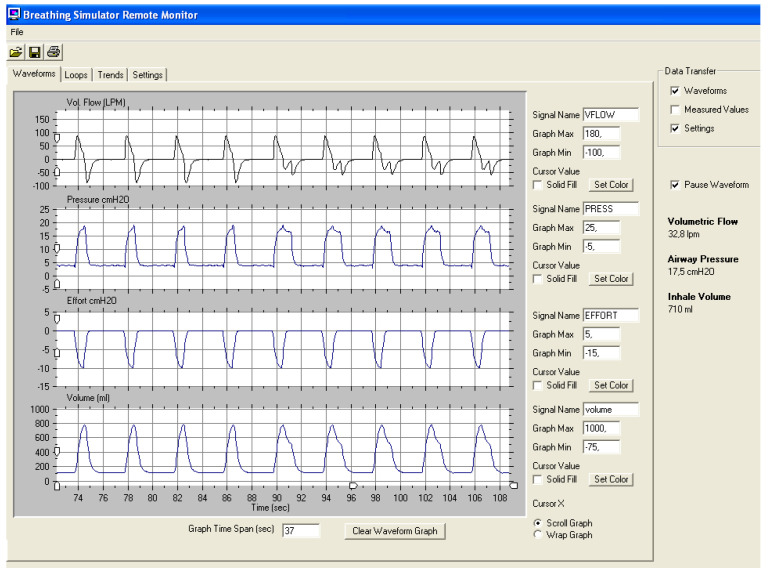
Bench experiment evaluating minimum inspiratory time. In the first half of the tracing, Ti min was not set, whereas in the second half was set at 1.3 s, without gaining any volume (fourth channel). Parameters: IPAP 18/EPAP 5, cycling criterion 10%.

These parameters are considered “safety cycling” parameters. In the case of maximum inspiratory time, it works as an inspiratory time limiter in the case of important leaks, when the flow cycling criterion would never be reached (or reached too late for the neural inspiratory time of the patient) leaving the ventilator inadequately in the inspiratory phase. By contrast, the function of the minimum inspiratory time seems more controversial: its main role according to some manufacturers would be to ensure adequate inspiratory phase time, with improvement in alveolar ventilation. In the experiment shown in Figure 3, the volume gain after adding a minimum inspiratory time is nearly zero, as the excess time occurs at the end of the flow-time curve when its area under the curve (volume) is smaller. Finally, in some models, it has been shown that the Ti min cannot be lower than the pressurization ramp, which could lead to a malfunction of the device [31].

#### 2.2.7. Rise Fall or Expiratory Ramp

It is present only in some ventilator models. It acts in a similar way to the inspiratory ramp but in the transition from inspiration to expiration, which may be more abrupt or slower. There are no studies in the literature to support its usefulness. It should be considered that the use of slower than usual ramps may tend to increase the inspiratory time.

## 3. Autotitration Algorithms

In general, autotitrating algorithms operate as closed-loop systems. In them, the ventilator takes continuously some decisions, modifying some parameters based on a measured or estimated variable.

### 3.1. Volume-Assured Pressure-Limited Modes

Within the pressure-limited modes, hybrid modes have emerged aiming to maintain an assured tidal volume, keeping at the same time the advantages of barometric ventilation. These modes adjust the pressure support within a preset range (IPAP min and IPAP max) that will never be exceeded even if the programmed volume is not reached (interbreath) [13]. There is a less commonly used volume-assured pressure support mode (intrabreath) that prolongs inspiratory time at constant flow until the programmed volume is reached. The main problem with this mode is the excessive prolongation of inspiratory time [32], especially in obstructive patients.

One of the main problems in these systems is the response to an estimated and not directly measured parameter, such as the tidal volume, especially in a single limb configuration. Tidal volume is usually estimated by determining the leakage and subtracting it from the total flow at the ventilator outlet [33]. The determination of leakage is usually calculated from a point in the respiratory cycle where the patient’s flow is zero. Hence the transition from expiration to inspiration is used. From that point, leak values are extrapolated over the entire cycle. This algorithm may have some inaccuracies, especially in the case of asymmetric or non-linear leakage [34], which can significantly influence the performance of these autotitration algorithms [35].

The most used interbreath autotitration algorithms are the following:

#### 3.1.1. Average Volume-Assured Pressure Support (AVAPS, Phillips Respironics Murrysville, PA)

In this mode, the pressure support level changes within a minimum and maximum range to achieve a clinician-specified average assured tidal volume [36]. The rate of change in the pressure support was constant in early models (around 0.5–1 cm H_2_O/min), but it can be modified in the newer models, up to 5 cmH_2_O/min.

Some clinical benefits have been reported in this modality. In a randomized study, Magdy et al. demonstrated reduced PaCO_2_ (0.6 mm Hg) and increased PaO_2_ (59.6 vs. 57.7 mm Hg) in the AVAPS-treated group versus conventional pressure support after 6 months of treatment. They also demonstrated an increase in the 6-min walking test and an improvement in quality of life indices. [37] However, a recent meta-analysis, which included eight trials, concluded that there was no difference in clinical outcomes with respect to pressure support mode [38].

#### 3.1.2. The “Intelligent Volume Assured Pressure Support” Mode (IVAPS, Resmed San Diego, CA)

This is a modality that is also based on the calculation of the target alveolar ventilation using height as the reference to subtract anatomical dead space. Alveolar ventilation is the objective to be assured mainly by means of changes in pressure support. In addition, this system incorporates an algorithm for modifying the respiratory rate, also based on the alveolar ventilation to be achieved. This modality has also been tested in clinical practice in several studies. Nilius et al. demonstrated significantly higher pressure support values using iVAPS, but no difference in mean nocturnal transcutaneous CO_2_ pressure [39]. A trend towards more restful sleep was documented with iVAPS by Ekkerkamp et al., but without further improvements in gas exchange [40]. Better overnight adherence in naïve patients was also reported [41]. Finally, another study found non inferiority in nocturnal gas exchange compared with conventional pressure support [42].

In summary, volume-assured pressure support systems appear to be non-inferior to conventional pressure support, although the evidence in favor of their use still seems insufficient. In fact, a recent European task force on chronic ventilation in stable COPD recommended the use of fixed pressure support as the first-choice ventilator mode, given the uncertainties on the effect of autotitrating modes and the heterogeneity of autotitration algorithms available in the market [43]. In any case it seems advisable, if such a modality is chosen, that the lower limit of IPAP selected should not be too low to avoid pressure support decreases due to an error in the estimation of tidal volume in the presence of asymmetric leaks [35].

### 3.2. Auto-EPAP Systems

EPAP self-titration algorithms have been developed mainly for two purposes. The first is the maintenance of upper airway patency, similar to the performance of automatic CPAP systems. The second, which is pending commercialization, self-titrates EPAP to treat expiratory flow limitation (EFL).

#### 3.2.1. Autotitration Algorithms to Maintain Upper Airway Patency

There is an even more sophisticated variant of the hybrid modes, which also includes automatic titration of EPAP to maintain airway patency. These obstructions are determined by different mechanisms depending on the manufacturer. For example, in the AVAPS-AE mode (Philips Respironics), the patency of the upper airway is determined by means of the forced oscillation technique. Briefly, the ventilator sends out pressure oscillations of about 2 cm H_2_O amplitude at a frequency of 5 Hz at predetermined intervals. When the upper airway is open, the pressure oscillations are accompanied by effective flow. In contrast, when the airway is closed, there is no effective flow during the pressure oscillations. When the ventilator detects an obstruction, there is an increase in EPAP and vice versa, when the upper airway is open, the EPAP level decreases. Similarly, to the volume assured pressure support modes, the maximum and minimum EPAP values must be set at the ventilator. The main technical issue with that approach would appear when the obstructions do not match with the oscillation periods.

**Figure 4:**
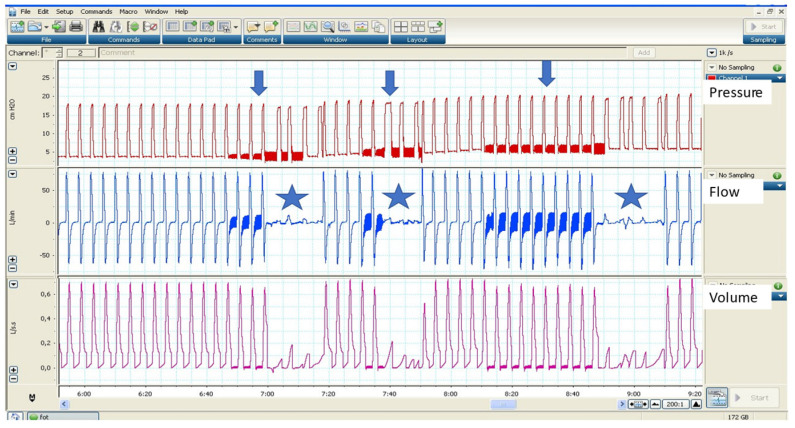
Example of AVAPS AE in a bench test. Observe the random intervals at which the forced oscillation technique is activated (arrows). When the system detects an obstruction (stars), there is an increase in EPAP.

Other manufacturers use the maximum flow to determine upper airway obstructions and autotitration EPAP algorithms. Figure 4 shows an example of AVAPS AE with the forced oscillation method.

As in the previous modes, EPAP autotitration for the treatment of upper airway obstructions (UAO) should show clinical benefits. In a randomized controlled study, Patout et al. demonstrated that AVAPS AE did not show additional benefits in pulmonary gas exchange and sleep parameters compared to conventional pressure support in a cohort of patients with obesity-associated hypoventilation syndrome [44]. Orr et al. demonstrated that the addition of automatic EPAP to the iVAPS mode was not inferior to manual EPAP titration in the iVAPS mode without the Auto-EPAP algorithm [45]. It is also interesting to note that there are important differences between the different algorithms in responding to simulated events, as recently demonstrated by Delorme et al. Periodic decreases in flow may correspond to different entities such as total or partial closure (hypopnoea) of the airway or be of central origin. The four ventilators, with different upper airway patency determination algorithms, showed different performances under the simulated conditions [46]. The concomitant presence of unintentional leakage may also contribute to undesired responses of auto-EPAP algorithms [47].

**Figure 5:**
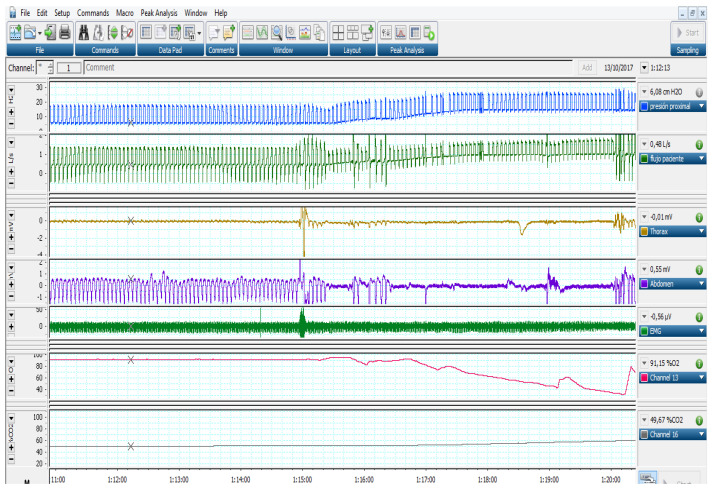
Example of inappropriate response of an automatic mode in a patient with UAO without effort (glottic closure). Observe the absence of movement in belts (Channels 3 and 4). In this case, the increase in pressure (Channel 1) was associated with a profound desaturation (Channel 6) and transcutaneous CO_2_ increase (Channel 7).

As a final reflection on the application of autoEPAP algorithms for UAO, it is worth remembering that not all of them respond to EPAP increase. Jounieaux et al. described more than 20 years ago the existence of mechanisms of upper airway obstruction at the glottis level due to high pressures or hyperventilation [48,49,50]. As a main feature, these obstructions show no signs of struggle in the thoracic and abdominal belts [51]. The autotitration EPAP algorithms cannot distinguish the level of obstruction and may respond inappropriately to the physiopathology of these obstructions. A clinical example is shown in Figure 5.

#### 3.2.2. Autotitration Algorithms to Treat Expiratory Flow Limitation (EFL)

EFL is defined as the inability to increase expiratory flow despite an increase in the pressure exerted by the expiratory muscles and should not be misinterpreted as the flow limitation that occurs in partial upper airway closures. It was defined from the findings of the seminal studies from Fry and Hyatt [52,53,54]. It is a phenomenon that occurs mainly in patients with COPD and is considered a poor prognostic factor in the evolution of the disease [55]. It may also occur in a small percentage of patients with obesity hypoventilation syndrome [56]. EFL leads to other pathophysiological phenomena that worsen pulmonary mechanics and pulmonary gas exchange, such as air trapping or increased intrinsic PEEP [57].

The techniques to detect EFL in mechanically ventilated patients have been the decrease in applied PEEP with expiratory flow monitoring or to apply a negative expiratory pressure (NEP) in the spontaneously ventilated patient [58].

For some years now, the use of FOT has been postulated as an alternative mechanism to determine the presence of EFL in patients on NIV [59,60]. The basis for its diagnosis is the difference in reactance between inspiration and expiration in response to the oscillations continuously delivered by the ventilator. Zannin et al. demonstrated a good correlation with the Mead and Whiterberger [61] technique, considered the gold standard for the diagnosis of EFL [62].

Concerning treatment, in mechanically ventilated patients, the classical mechanism used to treat EFL has been to increase eternal PEEP (EPAP) [63]. From all this background, an algorithm has been implemented on prototype ventilators (Philips Respironics) for the detection and management of EFL using FOT as a diagnostic tool and EPAP autotitration as a therapeutic mode. This ventilator model is still awaiting commercialization. In previous studies, Suh et al. demonstrated that EPAP autotitration in ventilated patients was associated with lower transdiaphragmatic pressure and decreased neural ventilatory drive [64]. In a randomized controlled study, it was shown that the use of EPAP autotitration resulted in a decrease in ineffective efforts and an improvement in hypercapnia [65]. Finally, McKenzie et al. demonstrated high variability in the presence of EFL in COPD patients [66]. Despite these promising results, and pending the commercialization of definitive firmware, long-term studies are needed to demonstrate the impact of this ventilatory modality on the survival or quality of life of ventilated patients.

## Data Availability

Not applicable.

## References

[1] Lassen H.C. (1953). A preliminary report on the 1952 epidemic of poliomyelitis in Copenhagen with special reference to the treatment of acute respiratory insufficiency. Lancet.

[2] Bach J.R., Alba A., Mosher R., Delaubier A. (1987). Intermittent positive pressure ventilation via nasal access in the management of respiratory insufficiency. Chest.

[3] Bach J.R., Alba A.S., Shin D. (1989). Management alternatives for post-polio respiratory insufficiency. Assisted ventilation by nasal or oral-nasal interface. Am. J. Phys. Med. Rehabil..

[4] Melloni B., Mounier L., Laaban J.-P., Chambellan A., Foret D., Muir J.-F. (2018). Home-Based Care Evolution in Chronic Respiratory Failure between 2001 and 2015 (Antadir Federation Observatory). Respir. Int. Rev. Thorac. Dis..

[5] Scott J.B. (2019). Ventilators for Noninvasive Ventilation in Adult Acute Care. Respir. Care.

[6] Scala R., Naldi M. (2008). Ventilators for noninvasive ventilation to treat acute respiratory failure. Respir. Care.

[7] Luján M., Peñuelas Ó., Cinesi Gómez C., García-Salido A., Moreno Hernando J., Romero Berrocal A., Gutiérrez Ibarluzea I., Masa Jiménez J.F., Mas A., Carratalá Perales J.M. (2020). Summary of Recommendations and Key Points of the Consensus of Spanish Scientific Societies (SEPAR, SEMICYUC, SEMES; SECIP, SENEO, SEDAR, SENP) on the Use of Non-Invasive Ventilation and High-Flow Oxygen Therapy with Nasal Cannulas in Adult, Pediatric, and Neonatal Patients with Severe Acute Respiratory Failure. Arch. Bronconeumol..

[8] Luján M., Sogo A., Monsó E. (2012). Home mechanical ventilation monitoring software: Measure more or measure better?. Arch. Bronconeumol..

[9] Johnson K.G. (2022). APAP, BPAP, CPAP, and New Modes of Positive Airway Pressure Therapy. Adv. Exp. Med. Biol..

[10] Gregoretti C., Navalesi P., Ghannadian S., Carlucci A., Pelosi P. (2013). Choosing a ventilator for home mechanical ventilation. Breathe.

[11] Lloyd-Owen S.J., Donaldson G.C., Ambrosino N., Escarabill J., Farre R., Fauroux B., Robert D., Schoenhofer B., Simonds A.K., Wedzicha J.A. (2005). Patterns of home mechanical ventilation use in Europe: Results from the Eurovent survey. Eur. Respir. J..

[12] Georgopoulos D., Prinianakis G., Kondili E. (2006). Bedside waveforms interpretation as a tool to identify patient-ventilator asynchronies. Intensive Care Med..

[13] Rabec C., Rodenstein D., Leger P., Rouault S., Perrin C., Gonzalez-Bermejo J. (2011). SomnoNIV group Ventilator modes and settings during non-invasive ventilation: Effects on respiratory events and implications for their identification. Thorax.

[14] Contal O., Adler D., Borel J.-C., Espa F., Perrig S., Rodenstein D., Pépin J.-L., Janssens J.-P. (2013). Impact of different backup respiratory rates on the efficacy of noninvasive positive pressure ventilation in obesity hypoventilation syndrome: A randomized trial. Chest.

[15] Sinderby C., Navalesi P., Beck J., Skrobik Y., Comtois N., Friberg S., Gottfried S.B., Lindström L. (1999). Neural control of mechanical ventilation in respiratory failure. Nat. Med..

[16] Yonis H., Crognier L., Conil J.-M., Serres I., Rouget A., Virtos M., Cougot P., Minville V., Fourcade O., Georges B. (2015). Patient-ventilator synchrony in Neurally Adjusted Ventilatory Assist (NAVA) and Pressure Support Ventilation (PSV): A prospective observational study. BMC Anesthesiol..

[17] Goulet R., Hess D., Kacmarek R.M. (1997). Pressure vs flow triggering during pressure support ventilation. Chest.

[18] Aslanian P., El Atrous S., Isabey D., Valente E., Corsi D., Harf A., Lemaire F., Brochard L. (1998). Effects of flow triggering on breathing effort during partial ventilatory support. Am. J. Respir. Crit. Care Med..

[19] Innovation and Technology|ResMed. https://www.resmed.com/ap/en/healthcare-professional/products/innovation-and-technology/ventilation-innovation-and-technology.html.

[20] Ferreira J.C., Chipman D.W., Hill N.S., Kacmarek R.M. (2009). Bilevel vs ICU ventilators providing noninvasive ventilation: Effect of system leaks: A COPD lung model comparison. Chest.

[21] Carteaux G., Lyazidi A., Cordoba-Izquierdo A., Vignaux L., Jolliet P., Thille A.W., Richard J.-C.M., Brochard L. (2012). Patient-ventilator asynchrony during noninvasive ventilation: A bench and clinical study. Chest.

[22] Ueno Y., Nakanishi N., Oto J., Imanaka H., Nishimura M. (2011). A bench study of the effects of leak on ventilator performance during noninvasive ventilation. Respir. Care.

[23] Prinianakis G., Kondili E., Georgopoulos D. (2003). Effects of the flow waveform method of triggering and cycling on patient-ventilator interaction during pressure support. Intensive Care Med..

[24] Gonzalez-Bermejo J., Janssens J.-P., Rabec C., Perrin C., Lofaso F., Langevin B., Carlucci A., Lujan M. (2019). SomnoNIV group Framework for patient-ventilator asynchrony during long-term non-invasive ventilation. Thorax.

[25] Lalmolda C., Flórez P., Grimau C., Larrosa R., Corral M., Sayas J., Luján M. (2021). A bench-to-bedside study about trigger asynchronies induced by the introduction of external gas into the non-invasive mechanical ventilation circuit. Sci. Rep..

[26] US Patent Application for A System and Method for Synchronization of Breathing in a Mechanical Ventilator Patent Application (Application #20160008559 Issued 14 January 2016)—Justia Patents Search. https://patents.justia.com/patent/20160008559.

[27] Zhu K., Rabec C., Gonzalez-Bermejo J., Hardy S., Aouf S., Escourrou P., Roisman G. (2017). Combined effects of leaks, respiratory system properties and upper airway patency on the performance of home ventilators: A bench study. BMC Pulm. Med..

[28] Battisti A., Tassaux D., Janssens J.-P., Michotte J.-B., Jaber S., Jolliet P. (2005). Performance characteristics of 10 home mechanical ventilators in pressure-support mode: A comparative bench study. Chest.

[29] Delgado C., Romero J.E., Puig J., Izquierdo A., Ferrando C., Belda F.J., Soro M. (2017). Performance of the New Turbine Mid-Level Critical Care Ventilators. Respir. Care.

[30] Lalmolda C., Flórez P., Corral M., Hernández Voth A., Grimau C., Sayas J., Luján M. (2022). Does the Efficacy of High Intensity Ventilation in Stable COPD Depend on the Ventilator Model? A Bench-to-Bedside Study. Int. J. Chron. Obstruct. Pulmon. Dis..

[31] Gonzalez-Bermejo J., Rabec C., Janssens J.P., Perrin C., Langevin B., Pepin J.L., Rodenstein D. (2013). Noninvasive ventilation inefficacy due to technically incompatible ventilator settings. Intensive Care Med..

[32] Amato M.B., Barbas C.S., Bonassa J., Saldiva P.H., Zin W.A., de Carvalho C.R. (1992). Volume-assured pressure support ventilation (VAPSV). A new approach for reducing muscle workload during acute respiratory failure. Chest.

[33] Luján M., Lalmolda C., Ergan B. (2019). Basic Concepts for Tidal Volume and Leakage Estimation in Non-Invasive Ventilation. Turk. Thorac. J..

[34] Sogo A., Montanyà J., Monsó E., Blanch L., Pomares X., Lujàn M. (2013). Effect of dynamic random leaks on the monitoring accuracy of home mechanical ventilators: A bench study. BMC Pulm. Med..

[35] Luján M., Sogo A., Grimau C., Pomares X., Blanch L., Monsó E. (2015). Influence of dynamic leaks in volume-targeted pressure support noninvasive ventilation: A bench study. Respir. Care.

[36] Briones Claudett K.H., Briones Claudett M., Chung Sang Wong M., Nuques Martinez A., Soto Espinoza R., Montalvo M., Esquinas Rodriguez A., Gonzalez Diaz G., Grunauer Andrade M. (2013). Noninvasive mechanical ventilation with average volume assured pressure support (AVAPS) in patients with chronic obstructive pulmonary disease and hypercapnic encephalopathy. BMC Pulm. Med..

[37] Magdy D.M., Metwally A. (2020). Effect of average volume-assured pressure support treatment on health-related quality of life in COPD patients with chronic hypercapnic respiratory failure: A randomized trial. Respir. Res..

[38] Huang X.A., Du Y.P., Li L.X., Wu F.F., Hong S.Q., Tang F.X., Ye Z.Q. (2019). Comparing the effects and compliance between volume-assured and pressure support non-invasive ventilation in patients with chronic respiratory failure. Clin. Respir. J..

[39] Nilius G., Katamadze N., Domanski U., Schroeder M., Franke K.-J. (2017). Non-invasive ventilation with intelligent volume-assured pressure support versus pressure-controlled ventilation: Effects on the respiratory event rate and sleep quality in COPD with chronic hypercapnia. Int. J. Chron. Obstruct. Pulmon. Dis..

[40] Ekkernkamp E., Storre J.H., Windisch W., Dreher M. (2014). Impact of intelligent volume-assured pressure support on sleep quality in stable hypercapnic chronic obstructive pulmonary disease patients: A randomized, crossover study. Respir. Int. Rev. Thorac. Dis..

[41] Kelly J.L., Jaye J., Pickersgill R.E., Chatwin M., Morrell M.J., Simonds A.K. (2014). Randomized trial of “intelligent” autotitrating ventilation versus standard pressure support non-invasive ventilation: Impact on adherence and physiological outcomes. Respirology.

[42] Oscroft N.S., Chadwick R., Davies M.G., Quinnell T.G., Smith I.E. (2014). Volume assured versus pressure preset non-invasive ventilation for compensated ventilatory failure in COPD. Respir. Med..

[43] Ergan B., Oczkowski S., Rochwerg B., Carlucci A., Chatwin M., Clini E., Elliott M., Gonzalez-Bermejo J., Hart N., Lujan M. (2019). European Respiratory Society guidelines on long-term home non-invasive ventilation for management of COPD. Eur. Respir. J..

[44] Patout M., Gagnadoux F., Rabec C., Trzepizur W., Georges M., Perrin C., Tamisier R., Pépin J.-L., Llontop C., Attali V. (2020). AVAPS-AE versus ST mode: A randomized controlled trial in patients with obesity hypoventilation syndrome. Respirology.

[45] Orr J.E., Coleman J., Criner G.J., Sundar K.M., Tsai S.C., Benjafield A.V., Crocker M.E., Willes L., Malhotra A., Owens R.L. (2019). Automatic EPAP intelligent volume-assured pressure support is effective in patients with chronic respiratory failure: A randomized trial. Respirology.

[46] Delorme M., Leroux K., Leotard A., Boussaid G., Prigent H., Louis B., Lofaso F. (2023). Noninvasive Ventilation Automated Technologies: A Bench Evaluation of Device Responses to Sleep-Related Respiratory Events. Respir. Care.

[47] Fasquel L., Yazdani P., Zaugg C., Barras A., Michotte J.-B., Correvon N., Contal O. (2023). Impact of Unintentional Air Leaks on Automatic Positive Airway Pressure Device Performance in Simulated Sleep Apnea Events. Respir. Care.

[48] Jounieaux V., Aubert G., Dury M., Delguste P., Rodenstein D.O. (1995). Effects of nasal positive-pressure hyperventilation on the glottis in normal sleeping subjects. J. Appl. Physiol..

[49] Jounieaux V., Rodenstein D.O., Mahjoub Y. (2020). On Happy Hypoxia and on Sadly Ignored “Acute Vascular Distress Syndrome” in Patients with COVID-19. Am. J. Respir. Crit. Care Med..

[50] Jounieaux V., Rodenstein D.O. (2019). Glottic patency during noninvasive ventilation in patients with chronic obstructive pulmonary disease. Respir. Physiol. Neurobiol..

[51] Gonzalez-Bermejo J., Perrin C., Janssens J.P., Pepin J.L., Mroue G., Léger P., Langevin B., Rouault S., Rabec C., Rodenstein D. (2012). Proposal for a systematic analysis of polygraphy or polysomnography for identifying and scoring abnormal events occurring during non-invasive ventilation. Thorax.

[52] Fry D.L., Ebert R.V., Stead W.W., Brown C.C. (1954). The mechanics of pulmonary ventilation in normal subjects and in patients with emphysema. Am. J. Med..

[53] Hyatt R.E., Schilder D.P., Fry D.L. (1958). Relationship between maximum expiratory flow and degree of lung inflation. J. Appl. Physiol..

[54] Hyatt R.E. (1983). Expiratory flow limitation. J. Appl. Physiol..

[55] Dean J., Kolsum U., Hitchen P., Gupta V., Singh D. (2017). Clinical characteristics of COPD patients with tidal expiratory flow limitation. Int. J. Chron. Obstruct. Pulmon. Dis..

[56] Anderson M.R., Shashaty M.G.S. (2021). Impact of Obesity in Critical Illness. Chest.

[57] Junhasavasdikul D., Telias I., Grieco D.L., Chen L., Gutierrez C.M., Piraino T., Brochard L. (2018). Expiratory Flow Limitation During Mechanical Ventilation. Chest.

[58] Akita T., Shirai T., Mori K., Shimoda Y., Suzuki T., Hayashi I., Noguchi R., Mochizuki E., Sakurai S., Saigusa M. (2016). Association of the forced oscillation technique with negative expiratory pressure in COPD. Respir. Physiol. Neurobiol..

[59] Dellacà R.L., Rotger M., Aliverti A., Navajas D., Pedotti A., Farré R. (2006). Noninvasive detection of expiratory flow limitation in COPD patients during nasal CPAP. Eur. Respir. J..

[60] Dellacà R.L., Duffy N., Pompilio P.P., Aliverti A., Koulouris N.G., Pedotti A., Calverley P.M.A. (2007). Expiratory flow limitation detected by forced oscillation and negative expiratory pressure. Eur. Respir. J..

[61] Mead J., Whittenberger J.L. (1954). Evaluation of airway interruption technique as a method for measuring pulmonary airflow resistance. J. Appl. Physiol..

[62] Zannin E., Chakrabarti B., Govoni L., Pompilio P.P., Romano R., Calverley P.M.A., Dellacà R.L. (2019). Detection of Expiratory Flow Limitation by Forced Oscillations during Noninvasive Ventilation. Am. J. Respir. Crit. Care Med..

[63] Monteiro M.B., Berton D.C., Moreira M.A.F., Menna-Barreto S.S., Teixeira P.J.Z. (2012). Effects of expiratory positive airway pressure on dynamic hyperinflation during exercise in patients with COPD. Respir. Care.

[64] Suh E.-S., Pompilio P., Mandal S., Hill P., Kaltsakas G., Murphy P.B., Romano R., Moxham J., Dellaca R., Hart N. (2020). Autotitrating external positive end-expiratory airway pressure to abolish expiratory flow limitation during tidal breathing in patients with severe COPD: A physiological study. Eur. Respir. J..

[65] Zannin E., Milesi I., Porta R., Cacciatore S., Barbano L., Trentin R., Fanfulla F., Vitacca M., Dellacà R.L. (2020). Effect of nocturnal EPAP titration to abolish tidal expiratory flow limitation in COPD patients with chronic hypercapnia: A randomized, cross-over pilot study. Respir. Res..

[66] McKenzie J., Nisha P., Cannon-Bailey S., Cain C., Kissel M., Stachel J., Proscyk C., Romano R., Hardy B., Calverley P.M.A. (2021). Overnight variation in tidal expiratory flow limitation in COPD patients and its correction: An observational study. Respir. Res..

